# Agreement between children with long-term health conditions and their primary caregivers on reports of perceived participation

**DOI:** 10.3389/fresc.2023.1123651

**Published:** 2023-06-07

**Authors:** Hong Zheng, Juan Bornman, Mats Granlund, Yue Zhao, Karina Huus

**Affiliations:** ^1^ CHILD Research Group, Swedish Institute of Disability Research, School of Health and Welfare, Jönköping University, Jönköping, Sweden; ^2^ School of Nursing, Tianjin Medical University, Tianjin, China; ^3^ Centre for Augmentative and Alternative Communication, University of Pretoria, Pretoria, South Africa

**Keywords:** agreement, participation, long-term health conditions, child reports, proxy reports

## Abstract

**Background:**

There is limited knowledge regarding the perceived participation of children with long-term health conditions in everyday activities. Children may have perceptions that differ from those of their primary caregivers. It is unclear whether children and caregivers rate their participation in everyday situations in the same way.

**Objectives:**

We aimed to explore the level of agreement pertaining to perceived participation (attendance and involvement) and examine whether differences exist in the rank order of activities selected as the three most important between reports from children with long-term health conditions and their primary caregivers.

**Methods:**

The simplified Chinese version of the Picture My Participation (PMP-C; Simplified) was used in an interview with children with long-term health conditions; meanwhile, their primary caregivers finished the questionnaire independently. Data were analyzed using Wilcoxon tests, weighted kappa values, and Spearman's rank order correlation.

**Results:**

Children with long-term health conditions reported significantly lower attendance scores for six activity items (*p* < 0.05) and higher involvement scores for two activity items (*p* < 0.05) than their primary caregivers did. An overall slight to fair agreement in perceived participation was found at the child–caregiver dyad level, though differences in dyads were observed. A strong correlation was identified between the rank order of the most important activities for both groups (*r* = 0.81).

**Conclusions:**

Differences may exist between the perceived participation of children with long-term health conditions, as reported by primary caregivers and the children themselves. The findings highlight that children with long-term health conditions exhibit unique views with respect to their perceived participation and have to be asked regarding their perceptions themselves.

## 1. Introduction

Considering long-term health conditions’ chronic nature, assessing how long-term health conditions affect afflicted children's daily lives is important. Participation—a critical health-related behavior among children with long-term health conditions—has been the focus of varied recent research initiatives worldwide. According to previous research, children with long-term health conditions—compared to their healthy peers—are restricted with respect to participation in daily activities because of their lower physical fitness levels, negative side effects of the medication, increased frequency of clinical visits or hospital admissions, and less free time (1, 2). Furthermore, family members, the child's school, and the community often overprotect children with long-term health conditions owing to factors such as potential bullying or risk of infection, which may contribute to preventing children from participating in daily activities (3, 4). Therefore, participation is a crucial goal and outcome of rehabilitation in children with long-term health conditions (5).

Long-term health conditions are defined as health problems lasting for more than 3 months that cannot be resolved spontaneously and require hospitalization, home healthcare, and/or extensive medical care (6). Long-term health conditions negatively impact children's regular activities, health, and wellbeing (7). Previous study implies that the effect is not diagnosis-specific, although more research evidence is needed (8). Globally, long-term health conditions are common among children and adolescents, particularly in low- and middle-income countries (LMICs) (9). The estimated prevalence of children with long-term health conditions has been increasing over the past 30 years; notably, 14% of the children in China are affected (10). Additionally, the complexity and severity of long-term health conditions, such as leukemia and congenital heart disease, continue increasing, especially in children who bear the greatest burden of these chronic diseases. Furthermore, the majority of these children are likely to have a lifetime of managing their long-term conditions, which may affect their health outcomes in adulthood as well as their future opportunities and quality of life (11).

Investigating a child's own perceptions of participation can provide a more comprehensive description of long-term health conditions’ impact on children's health and wellbeing. Therefore, researchers have highlighted the importance of obtaining participation assessments from the child's own perspective (12, 13). Moreover, the United Nations Convention on the Rights of the Child (CRC) stresses the importance of children's own expressions in conjunction with decision-making in daily life (14). Children's self-ratings of participation may play a key role in offering them an opportunity to express their own opinions on how they experience their individual participation in specific activities, thereby providing a picture of the child's actual life as well as the aspects that must be changed (15).

However, to obtain a more holistic view of participation among children, some researchers have underscored the need to collect information from both the child's and proxy's perspectives (typically, a trusted adult, such as the primary caregiver) as a mechanism for gaining information regarding the family as a unit. A comprehensive assessment of the participation of children with long-term health conditions is likely to include reports from both children and caregivers. According to some authors, children are considered unreliable interviewees who lack the cognitive and linguistic skills required to understand and answer questionnaires, which depend on the children's age and health conditions (16). As far as young children and those with long-term health conditions are concerned, proxy ratings can be considered substitutes for children's reports and provide important information regarding children's choices and perceptions (17). Hence, caregivers’ and children's ratings supplement each other and together provide a more comprehensive picture of how children's long-term health conditions affect their perceived participation and everyday life.

Participation is a complex and widely discussed concept (18, 19). Among the main models is the biopsychosocial model included in the World Health Organization (WHO) “International Classification of Functioning, Disability and Health” (ICF; WHO, 2001) and its child and youth version (ICF-CY; WHO, 2007). The ICF highlights participation as “involvement in life situations.” In a systematic review, Imms et al. (20) emphasized the family of participation-related constructs (FPRC) as a different model of participation in children based on ICF. Within the FPRC, participation is operationally defined as a separating concept from life situations in which participation occurs, and skills used in the activity (21). It is important to develop parallel tools to explore participation in any clinical or research endeavor. As participation attendance and/or involvement may be assessed as either an outcome or a process, the FPRC identifies participation as divided into the following two essential components: attendance, described as “being there” in an activity and can be assessed as frequency, and involvement, an experience of participation while attending and can be measured as the perceived involvement level (20, 22). This conceptualization of participation can be applied in any activity and/or context in children of any level of competence (23).

To the best of our knowledge, due to cultural diversity, there is a lack of knowledge regarding participation in daily activities for children with long-term health conditions in China. Accurate and deep information regarding the participation of children with long-term health conditions in China would be an urgent requirement to be investigated. Additionally, less is known regarding the similarities and differences in participation experiences between children with long-term health conditions and their caregivers. Reports from South Africa (15) and Taiwan (24) indicate that the perceptions of children and primary caregivers only partly agree in terms of both the frequency of participation and the importance assigned to activities. Therefore, our study aims to explore the level of agreement of perceived participation (attendance and involvement) and examine whether differences exist in the rank order of activities selected as the three most important between reports from children with long-term health conditions and their primary caregivers.

## 2. Materials and method

### 2.1. Participants

The study employed a cross-sectional, exploratory design to elucidate the participation of children with long-term health conditions in China from their own—as well as from their caregivers’—perspectives. A convenience sample of 65 child–caregiver dyads was recruited from inpatient wards in two specialized hospitals in Tianjin. To be eligible, children had to be between 5 and 18 years of age with typical development and had a long-term health condition. Children and caregivers had to be able to read and write Chinese. Dyads were excluded if either the child or caregiver dyad presented any comorbid condition (e.g., physical or mental health challenges) other than congenital heart disease or leukemia that required treatment. Prior to data collection, an information letter was provided to potential participants. Participants were informed that they could withdraw from the study at any time without providing a reason; thereafter, an assent form was obtained from the child, and a written informed consent form was gained from the primary caregiver. The study was approved by the Research Ethics Committee of Tianjin Medical University. The data were collected between November 2018 and August 2019.

Of the 108 child–caregiver dyads, eight children were excluded owing to limited Chinese language skills, while 20 children were excluded owing to their serious health conditions, leaving 80 dyads that fulfilled the inclusion criteria. Of the 80 dyads, three children's primary caregivers did not provide consent for their children to participate, while 12 children chose not to participate. This resulted in 65 dyads. Attrition may reflect no direct benefit to families for participation or insufficient time and interest in the family for participation in this study. At the time of data collection, all 65 dyads were present and provided complete data.

### 2.2. Measures

#### 2.2.1. Health status assessment

A patient schedule was used to contact eligible participants. Feedback from doctors, nurses, primary caregivers, and children was important to ensure that the children were during the rehabilitation period and exhibited stable health conditions, thereby allowing them to attend this study.

#### 2.2.2. Ten questions questionnaire

The “ten questions” questionnaire (TQQ) is designed for children to detect neurological disabilities (25). The TQQ includes 10 items in a yes/no format to screen children for cognitive, sensory, and motor disabilities, as well as for epilepsy. Zero points indicate no problem at all. In our present study, the TQQ was completed by primary caregivers to exclude that their children had any existing neurological disability and to ensure that the children were qualified for this research.

#### 2.2.3. Simplified Chinese version of the Picture my Participation

The Picture My Participation (PMP) is a new self-report measure that was developed to assess perceived participation with respect to attendance and involvement in children between 5 and 21 years of age, especially in LMICs. PMP has been widely used in children with ID and has been translated into several languages, including Chinese (26, 27). The content validity and reliability of the simplified Chinese version of the Picture My Participation (PMP-C; simplified) have been explored in previous studies (28). Specifically, the PMP-C (simplified) has a good internal consistency (α = 0.80) and test–retest reliability (ICC = 0.89) (28). Furthermore, PMP-C (simplified) has been used in children with autism spectrum disorder in an ongoing project in China. Thus, the PMP-C (simplified) is identified as an appropriate instrument of participation in children in mainland China.

In the current study, two versions of the PMP-C (simplified) were used: PMP-C (simplified) for children (self-report) and PMP-C (simplified) for caregivers (proxy report).

The PMP-C (simplified) comprises the following three sections. The first section refers to demographic information; for the child, it focuses on gender, age, and type of community, while for the primary caregiver, it focuses on age, educational level, employment, relationship to the child, and number of people living in the house. Although some questions focused on the child, only the caregivers completed the demographic section. The second section includes 20 activity items of perceived participation in daily activities. For each activity item, the frequency of attendance is rated on a four-point Likert scale ranging from “1 = never” to “4 = always,” while the child's involvement in the activity during participation is rated on a three-point Likert scale ranging from “1 = not involved” to “3 = very involved.” Both children and caregivers completed the section. In the last section, participants are asked to choose three activities that they consider the most important to the child. Both the children and caregivers completed this section of the questionnaire.

### 2.3. Data collection and procedures

Child–caregiver dyads were contacted in the inpatient wards of two hospitals. A survey pack was provided to the identified families chosen by the researcher in a sealed envelope. The survey pack contained the following items: (a) the information letter with a reply slip to indicate consent for the primary caregivers and their child, (b) TQQ, and (c) PMP-C (simplified). Primary caregivers were asked to complete all survey formats individually under the researcher's supervision and then return them to the researcher. Any question with which the caregiver experienced difficulty was explained by the researcher.

Thereafter, the researcher read the information letter to the child to obtain assent to participate in this study from the children themselves. An environment was created wherein the caregiver and child could not influence each other in the process of accomplishing the surveys or interviews. Child–caregiver dyads completed the PMP-C (simplified) separately to ensure that no data contamination occurred. The Talking Mats framework—as a strategy to facilitate responses to the PMP-C (simplified)—was used in face-to-face structured interviews between recruited child participants and the researcher (29). Graphic symbols from Picture Communication Symbols (PCS) were also used by children as visual support for the PMP-C instrument.

Interviews were conducted using demographic questions. Subsequently, three trial items were introduced by the researcher to ensure the children's understanding of rating the activities’ frequency. The child participants were asked to rate their attendance by placing each of the visual images of the 20 activities in a column on the frequency mat to indicate the response that most accurately represented their participation frequency in the respective activities. Thereafter, the researcher explained a new Talking Mat, which was now related to the “involvement” of participation. Thereafter, the child participants need to rate their involvement by sorting each of the symbols representing the 20 activities according to their degree of involvement on the mat in the relevant column (column 1 = not involved and column 3 = very involved). The researcher recorded sequentially responses to attendance and involvement regarding each activity. Finally, the children need to select three activities, which are the most important to themselves with the help of picture symbols regarding the specific activities. Then, the children were asked to rank these three activities on the mat from most to least important for themselves to indicate the activities’ prioritization. The researcher encouraged the children to share their own stories to elicit their voices and perspectives regarding their perceived participation throughout the conversations. Each child participant spent approximately 20–30 min to complete the interview, depending on the child's ability to follow the ideas and respond.

### 2.4. Statistical analysis

Data were exported to IBM SPSS Statistics 21.0 program and analyzed. Descriptive statistics were used to summarize the demographic characteristics in terms of means, standard deviations (SDs), and frequencies. The TQQ scores were presented as the means and SDs.

Frequency scores for the two participation constructs (attendance and involvement) were calculated item by item in the activities chosen by children and primary caregivers. As scores are not in a normal distribution, the range, mean, and SD were used to summarize the rating scores. Wilcoxon tests were utilized for the values with abnormal distribution to compare the frequency scores for each activity at the item level between both cohorts.

The proportion of agreements and disagreements in the two participation constructs (attendance and involvement) were calculated in child–caregiver dyads on a four-point scale. Frequencies of attendance and involvement between children and primary caregivers were calculated by item-level weighted kappa values (Ƙ) and were presented on the four-point scale. Weighted kappa values (Ƙ) indicate different extents of the agreement, which is considered poor (<0.01), slight (0.01–0.20), fair (0.21–0.40), moderate (0.41–0.60), substantial (0.61–0.80), and nearly perfect (0.81–1.00) (12). The rank order of the 20 items was calculated based on the frequencies of the items chosen as the most important by the two subgroups (i.e., children and primary caregivers). The relationship between both groups on the frequencies of the most important items was analyzed by Spearman's rank order correlation.

## 3. Results

### 3.1. Participants

Overall, 65 children with long-term health conditions and their primary caregivers participated in this study. The participants were 24 boys and 41 girls, and the children were aged 5–18 years (M = 11.3 years, SD = 3.1). On the TQQ, the primary caregivers reported no problems related to disability in their children. The primary caregivers’ questionnaires were predominantly completed by mothers (*n* = 42), followed by fathers (*n* = 20), and others (*n* = 3; one aunt and two sisters). Their ages ranged from 20 to 52 years (M = 37.9 years, SD = 7.0). Most primary caregivers (69.2%) were unemployed, and 66.2% had an educational level of 10 years or lower as their highest qualification. Table 1 illustrates the dyads’ demographic characteristics and TQQ scores.

**Table 1:** Sociodemographic characteristics and scores of the TQQ (*n* = 65 dyads).

Variable	*n* (%)
Gender	
Girls	41 (63.1)
Boys	24 (36.9)
Age (years, mean ± SD)	11.3 ± 3.1
Community type	
Urban	27 (41.5)
Rural	38 (58.5)
Long-term health conditions	
Congenital heart disease	32 (49.2)
Leukemia	33 (50.8)
TQQ (mean ± SD)	0 (0)
Caregivers’ gender	
Women	45 (69.2)
Men	20 (30.8)
Caregivers’ age (years, mean ± SD)	37.9 ± 7.0
Relationship with the child	
Father	20 (30.8)
Mother	42 (64.6)
Grandmother	0 (0)
Other	3 (4.6)
Work status	
Employed full time	12 (18.5)
Employed part-time	8 (12.3)
Unemployed	45 (69.2)
Caregivers’ highest educational level	
Grade 10 or lower	43 (66.2)
Grade 12	13 (20.0)
Diploma	2 (3.1)
Bachelor's degree	7 (10.8)
Postgraduate degree	0 (0)
Received a social grant	
Yes	9 (13.8)
No	56 (86.2)

TQQ, “ten questions” questionnaire.

### 3.2. Comparison of the attendance scores of participation

Complete data on the attendance component of the PMP-C (simplified) were available for all 65 dyads. Table 2 presents the results of the comparison of the attendance scores between children and their primary caregivers. The attendance scores of children with long-term health conditions were significantly lower than that of their primary caregivers for the following six activity items: gathering supplies, meal preparation, caring for family, celebrations, playing with others, and shopping.

**Table 2:** Item-by-item comparison of attendance scores between children and their primary caregivers (*n* = 65 dyads).

Activity item in the Chinese version of Picture My Participation (simplified)	Children (*n* = 65)			Primary caregivers (*n* = 65)			
	Min–Max	Mean	SD	Min–Max	Mean	SD	*p*-value
Personal care	2.00–4.00	3.63	0.72	2.00–4.00	3.78	0.48	0.097
Family mealtime	2.00–4.00	3.74	0.59	1.00–4.00	3.71	0.61	0.870
My own health	1.00–4.00	2.98	0.80	1.00–4.00	3.18	0.90	0.121
Gathering supplies	1.00–4.00	2.29	0.82	1.00–4.00	2.74	0.83	0.002*
Meal preparation	1.00–4.00	1.92	0.84	1.00–4.00	2.40	0.90	0.001*
Cleaning at home	1.00–4.00	2.71	0.82	1.00–4.00	2.77	0.81	0.498
Caring for family	1.00–4.00	2.62	0.95	1.00–4.00	2.92	0.82	0.028*
Caring for animals/pets	1.00–4.00	1.88	1.04	1.00–4.00	1.98	1.08	0.457
Family time	1.00–4.00	3.23	0.86	1.00–4.00	3.46	0.71	0.063
Celebrations	1.00–4.00	2.58	0.92	1.00–4.00	2.92	0.82	0.019*
Playing with others	1.00–4.00	3.03	0.87	1.00–4.00	3.32	0.79	0.023*
Organized leisure	1.00–4.00	2.69	0.98	1.00–4.00	2.85	0.97	0.360
Quiet leisure	1.00–4.00	3.06	0.81	1.00–4.00	3.02	0.88	0.654
Spiritual activities	1.00–4.00	1.29	0.61	1.00–4.00	1.22	0.55	0.425
Shopping	1.00–4.00	2.63	0.88	1.00–4.00	2.97	0.77	0.016*
Social activities	1.00–4.00	1.95	0.94	1.00–4.00	2.25	1.00	0.072
Health center participation	1.00–4.00	2.46	0.71	1.00–4.00	2.32	0.85	0.710
Attending school	2.00–4.00	3.78	0.45	1.00–4.00	3.88	0.38	0.248
Trips and visits	1.00–4.00	1.60	0.79	1.00–4.00	1.71	0.88	0.357
Employment	1.00–3.00	1.29	0.55	1.00–3.00	1.31	0.58	0.767

* *p* < 0.05.

### 3.3. Comparison of the involvement scores of participation

All 65 dyads responded to the PMP-C (simplified) involvement scale. The results of the comparison of involvement scores between the children and their primary caregivers are presented in Table 3. The involvement scores of children with long-term health conditions were significantly higher than those of their primary caregivers for the items cleaning at home and quiet leisure.

**Table 3:** Item-by-item comparison of involvement scores between children and their primary caregivers (*n* = 65 dyads).

Activity item in the Chinese version of Picture My Participation (simplified)	Children (*n* = 65)			Primary caregivers (*n* = 65)			
	Min–Max	Mean	SD	Min–Max	Mean	SD	*p*-value
Personal care	1.00–3.00	2.65	0.57	1.00–4.00	2.71	0.52	0.425
Family mealtime	1.00–3.00	2.74	0.57	1.00–4.00	2.78	0.48	0.572
My own health	1.00–3.00	2.32	0.79	1.00–3.00	2.22	0.76	0.340
Gathering supplies	1.00–3.00	2.09	0.79	1.00–4.00	2.06	0.78	0.842
Meal preparation	1.00–3.00	1.86	0.81	1.00–3.00	1.86	0.81	0.878
Cleaning at home	1.00–3.00	2.23	0.81	1.00–3.00	1.98	0.76	0.041*
Caring for family	1.00–3.00	2.23	0.77	1.00–3.00	2.15	0.69	0.581
Caring for animals/pets	1.00–3.00	1.66	0.87	1.00–4.00	1.62	0.88	0.703
Family time	1.00–3.00	2.63	0.57	1.00–3.00	2.52	0.69	0.282
Celebrations	1.00–3.00	2.43	0.75	1.00–4.00	2.32	0.79	0.379
Playing with others	1.00–3.00	2.52	0.71	1.00–3.00	2.62	0.65	0.452
Organized leisure	1.00–3.00	2.34	0.80	1.00–4.00	2.28	0.80	0.607
Quiet leisure	1.00–3.00	2.52	0.69	1.00–3.00	2.22	0.76	0.012*
Spiritual activities	1.00–3.00	1.23	0.58	1.00–3.00	1.09	0.38	0.146
Shopping	1.00–3.00	2.29	0.74	1.00–3.00	2.29	0.74	0.960
Social activities	1.00–3.00	1.92	0.87	1.00–3.00	1.75	0.79	0.194
Health center participation	1.00–3.00	1.78	0.80	1.00–3.00	1.63	0.72	0.174
Attending school	1.00–3.00	2.75	0.56	1.00–3.00	2.75	0.53	0.957
Trips and visits	1.00–3.00	1.51	0.79	1.00–3.00	1.54	0.83	0.656
Employment	1.00–3.00	1.32	0.71	1.00–3.00	1.23	0.58	0.301

* *p* < 0.05.

### 3.4. Proportion of agreement and disagreement between children and their primary caregivers on children's attendance at daily activities

For the children's attendance of 20 items of activities, the proportion of agreement in perceptions of children and primary caregivers ranged from 29.2% to 86.2%, and that of disagreement ranged from 13.8% to 70.8%. The weighted kappa values ranged from 0.077 to 0.432 (Table 4).

**Table 4:** Proportion of agreement and disagreement of children and their primary caregivers on children's attendance of daily activities.

Activity item in the Chinese version of Picture My Participation (simplified)	Agree (%)	Disagree (%)	Weighted kappa	95% CI	*p*
Personal care	69.2	30.8	0.185	−0.045 to 0.415	0.052
Family mealtime	64.6	35.4	0.067	−0.175 to 0.309	0.503
My own health	40.0	60.0	0.221	0.055 to 0.387	0.010*
Gathering supplies	38.5	61.5	0.145	−0.003 to 0.293	0.054
Meal preparation	40.0	60.0	0.195	0.032 to 0.359	0.010*
Cleaning at home	55.4	44.6	0.432	0.276 to 0.589	0.000*
Caring for family	41.5	58.5	0.173	0.009 to 0.338	0.028*
Caring for animals/pets	46.2	53.8	0.297	0.113 to 0.481	0.001*
Family time	40.0	60.0	0.077	−0.098 to 0.251	0.394
Celebrations	36.9	63.1	0.155	−0.020 to 0.330	0.059
Playing with others	43.1	56.9	0.172	0.001 to 0.343	0.052
Organized leisure	44.6	55.4	0.285	0.109 to 0.462	0.001*
Quiet leisure	52.3	47.7	0.400	0.235 to 0.564	0.000*
Spiritual activities	69.2	30.8	0.170	−0.020 to 0.359	0.087
Shopping	52.3	47.7	0.183	−0.004 to 0.370	0.018*
Social activities	29.2	70.8	0.079	−0.094 to 0.252	0.360
Health center participation	38.5	61.5	0.109	−0.063 to 0.281	0.196
Attending school	86.2	13.8	0.102	−0.192 to 0.397	0.343
Trips and visits	56.9	43.1	0.360	0.160 to 0.559	0.000*
Employment	70.8	29.2	0.317	0.083 to 0.552	0.003*

*p < 0.05.

### 3.5. Proportion of agreement and disagreement between children and their primary caregivers on children's involvement in daily activities

For the children's involvement in 20 items of activities, the proportion of agreement in perceptions of children and their primary caregivers ranged from 38.5% to 78.5%, and that of disagreement ranged from 18.5% to 61.5%. The weighted kappa values ranged from 0.024 to 0.353 (Table 5).

**Table 5:** Proportion of agreement and disagreement of children and their primary caregivers on children's involvement in daily activities.

Activity item in the Chinese version of Picture My Participation (simplified)	Agree (%)	Disagree (%)	Weighted kappa	95% CI	*p*
Personal care	69.2	30.8	0.317	0.106 to 0.528	0.003*
Family mealtime	69.2	30.8	0.157	−0.059 to 0.372	0.120
My own health	52.3	47.7	0.309	0.110 to 0.508	0.001*
Gathering supplies	46.2	53.8	0.199	0.005 to 0.394	0.032*
Meal preparation	46.2	53.8	0.148	−0.059 to 0.354	0.132
Cleaning at home	46.2	53.8	0.223	0.038 to 0.407	0.015*
Caring for family	41.5	58.5	0.109	−0.072 to 0.290	0.243
Caring for animals/pets	60.0	40.0	0.339	0.139 to 0.539	0.001*
Family time	53.8	46.2	0.099	−0.118 to 0.316	0.328
Celebrations	52.3	47.7	0.210	0.003 to 0.417	0.030*
Playing with others	47.7	52.3	−0.071	−0.238 to 0.095	0.483
Organized leisure	49.2	50.8	0.193	−0.019 to 0.404	0.047*
Quiet leisure	46.2	53.8	0.140	−0.040 to 0.319	0.125
Spiritual activities	81.5	18.5	0.024	−0.158 to 0.205	0.805
Shopping	38.5	61.5	0.051	−0.137 to 0.239	0.601
Social activities	43.1	56.9	0.164	−0.038 to 0.366	0.094
Health center participation	49.2	50.8	0.122	−0.070 to 0.314	0.203
Attending school	67.7	32.3	−0.044	−0.221 to 0.134	0.677
Trips and visits	61.5	38.5	0.188	−0.040 to 0.416	0.083
Employment	78.5	21.5	0.353	0.076 to 0.631	0.001*

*p < 0.05.

### 3.6. Comparison of the rank order of activities on frequencies of the items chosen as most important

All 65 dyads completed the selection of the three most important activities from the 20 items of activities. Table 6 displays the results regarding frequencies, item by item, of how frequently the 20 activity items of PMP-C (simplified) were selected as important to attend—and to be involved in. Children with long-term health conditions selected all 20 items, while their primary caregivers chose only 19 items and the spiritual activities item was out of the selection. The items “personal care” and “attending school” were the most frequently selected activities for both groups.

**Table 6:** Rank order of activities regarding frequencies of items selected as most important.

Activity item in the Chinese version of Picture My Participation (simplified)	Children (*n* = 65)	Primary caregiver (*n* = 65)	All (*n* = 130)
	(%)	(%)	(%)
Personal care	11.3	13.8	12.6
Attending school	12.8	10.3	11.5
Family time	7.2	12.3	9.7
My own health	10.3	7.7	9.0
Playing with others	7.2	6.1	6.7
Cleaning at home	5.6	7.2	6.4
Family mealtime	6.7	4.6	5.6
Organized leisure	4.1	7.2	5.6
Caring for family	5.1	3.1	4.1
Quiet leisure	5.1	3.1	4.1
Trips and visits	4.6	3.1	3.8
Gathering supplies	2.1	4.6	3.3
Meal preparation	2.6	4.1	3.3
Shopping	4.6	2.1	3.3
Celebrations	2.6	3.6	3.1
Caring for animals/pets	3.6	2.1	2.8
Employment	2.1	2.1	2.1
Social activities	1.5	1.5	1.5
Health center participation	0.5	1.5	1.0
Spiritual activities	0.5	0	0.3
Total	100.0	100.0	100.0

Spearman's rank order correlation analysis revealed a strong correlation between the rank orders of the most important activities for both groups (*r* = 0.81). The result indicates that the children and caregivers chose similar activities as important to attend—and to be involved in.

## 4. Discussion

The present study aimed to explore the level of agreement of perceived participation (attendance and involvement) and examine whether differences exist in the three activities that were selected as most important when selections made by children with long-term health conditions and their primary caregivers were compared. Based on the results from this study of Chinese children with long-term health conditions, the items on the PMP-C (simplified) provided useful information for caregivers and clinicians to understand children's participation issues.

Overall, compared with their primary caregivers, children with long-term health conditions reported significantly lower scores on attendance in the activities of gathering supplies, caring for family, caring for animals/pets, celebrations, playing with others, and shopping and higher scores on involvement in cleaning at home and quiet leisure (*p* < 0.05). Our results suggest considerable differences in the perceived participation of children with long-term health conditions, as reported by primary caregivers vs. the children themselves. This finding is similar to those of earlier studies, which indicated a discrepancy in the perceptions of primary caregivers and children regarding perceived participation (15, 24). Therefore, it should be tempting to focus on interventions or categories to increase participation in children with long-term health conditions. The provision of family and community support to facilitate opportunities for children with long-term health conditions to participate in activities at home and in the community is an important way to promote participation for children with long-term health conditions. Meanwhile, when planning interventions aimed at increasing participation, collecting information regarding attendance from both the children and caregivers is important.

Specifically, a higher proportion of disagreements was found between ratings of children with long-term health conditions and primary caregivers with respect to social and family activities. Our results indicate that children with long-term health conditions and caregivers with a higher probability disagree with respect to social activities (e.g., organized leisure) and family activities (e.g., meal preparation). This is in line with Dada et al. (15), who found greater discordance between child and caregiver ratings for perceived participation in social activities. When children and caregivers evaluate the former's participation, they may draw on different values that could lead to discordance between raters (30). One possible explanation is that children living with long-term health conditions may assess their perceived participation depending on their subjective experience, whereas caregivers may vary in their awareness of the child's performance or in their sensitivity pertaining to the child's health concerns (31). Primary caregivers may be less likely to have a complete picture of the attendance and involvement of their children's participation in situations wherein they themselves were not present. Hence, our results highlight the importance of children's own perspectives in participant research, which can be linked to their rights.

The following important question arises: Why do caregivers overestimate their children's attendance of perceived participation, compared with the children's own rating? A possible explanation for caregivers’ overestimation of their children's participation may be related to the weak influence of children's medical conditions, especially in some seemingly simple and concrete activities. Caregivers may realize how well their children have adapted to their long-term health conditions and the notion that their children should be able to do everything that healthy children can do.

Regarding the involvement of children's perceived participation, higher scores were reported by children themselves than by their caregivers for the following items: cleaning at home and quiet leisure. The children rated their involvement as higher in these two activities than their caregivers. Nevertheless, our results indicate that in most activities, children with long-term health conditions and their primary caregivers rated their overall involvement at approximately the same level. Some researchers have suggested that children are more likely to rate themselves at the highest level and express what they can do (32). The finding supported that if children with long-term health conditions have the opportunity to participate in an activity, they perceive their involvement in activities just as healthy children do (32).

We also found an interesting result; overall, primary caregivers’ rank order of activities selected as the three most important was in line with the children's selection. In terms of perceived importance, children and primary caregivers selected similar activities of personal care, school, family time, and personal (my own) health as important. This finding indicates that both children and caregivers had similar expectations of activities in their lives. In this study, children with long-term health conditions presented a range of interests in all 20 activity items of the PMP-C. However, children with long-term health conditions may consider the three most important activities as activities as those that are typically likely to be done, that they like to do, or that they do frequently (24). On the contrary, primary caregivers who fulfill a primary role in guiding and protecting the child may prioritize selecting activities deemed to be beneficial or important for adult life. This result is in line with a previous study, which suggested that while caregivers may be valid proxies for their children, children with long-term health conditions may exhibit different views regarding their perceived participation (33, 34). Future studies can explore children's perceptions of the facilitators and barriers of the three most important activities to gain a more comprehensive understanding of participation. Future interventions should consider the activity preferences of children in developing programs, and accommodate individual needs, which can be beneficial for caregivers to provide guidance to improve children's participation and life skills.

A strength of our study is that it focused on comparing children's perceived participation (self-reports by children) and proxy ratings by caregivers. Moreover, this study explored the level of two constructs of participation—attendance and involvement—that have not been previously evaluated with children's self-ratings and compared the proportion of the three most important activities wherein child and caregiver dyads agreed.

However, this study exhibits several limitations. First, due to the small sample size, the sample was not sufficiently large to demonstrate the findings’ representativeness. Second, there is limited variability in diagnoses of long-term health conditions. The diversity of diagnoses need to be explored in further studies. The third limitation is that this study did not explore the effects of personal factors (i.e., child's self-efficacy, preference) and environmental factors on participation. Future studies should concentrate on the factors that may contribute to increasing participation.

## Data Availability

The raw data supporting the conclusions of this article will be made available by the authors. For further inquiries, contact the corresponding author.

## References

[1] Law M Anaby D Teplicky R Khetani MA Coster W Bedell G. Participation in the home environment among children and youth with and without disabilities. Br J Occup Ther. (2015) 76(2):58–66. 10.4276/030802213X13603244419112

[2] Van Brussel M van der Net J Hulzebos E Helders PJM Takken T. The Utrecht approach to exercise in chronic childhood conditions: the decade in review. Pediatr Phys Ther. (2011) 23(1):2–14. 10.1097/PEP.0b013e318208cb22 21304338

[3] Mei C Reilly S Reddihough D Mensah F Green J Pennington L Activities and participation of children with cerebral palsy: parent perspectives. Disabil Rehabil. (2015) 37(23):2164–73. 10.3109/09638288.2014.999164 25586796

[4] Riner WF Sellhorst SH. Physical activity and exercise in children with chronic health conditions. J Sport Health Sci. (2013) 2(1):12–20. 10.1016/j.jshs.2012.11.005

[5] Coster W Law M Bedell G Khetani M Cousins M Teplicky R. Development of the participation and environment measure for children and youth: conceptual basis. Disabil Rehabil. (2012) 34:238–45. 10.3109/09638288.2011.603017 21981404

[6] van der Lee JH Mokkink LB Grootenhuis MA Heymans HS Offringa M. Definitions and measurement of chronic health conditions in childhood: a systematic review. JAMA. (2007) 297(24):2741–51. 10.1001/jama.297.24.2741 17595275

[7] De Bock F Bosle C Graef C Oepen J Philippi H Urschitz MS. Measuring social participation in children with chronic health conditions: validation and reference values of the child and adolescent scale of participation (CASP) in the German context. BMC Pediatr. (2019) 19(1):125. 10.1186/s12887-019-1495-6 31018847 PMC6482577

[8] Wallander JL Varni JW. Effects of pediatric chronic physical disorders on child and family adjustment. J Child Psychol Psychiatry. (1998) 39(1):29–46. 9534085

[9] Kirk S Beatty S Callery P Gellatly J Milnes L Pryjmachuk S. The effectiveness of self-care support interventions for children and young people with long-term conditions: a systematic review. Child Care Health Dev. (2013) 39(3):305–24. 10.1111/j.1365-2214.2012.01395.x 22676438

[10] Zheng H Bornman J Granlund M Zhao Y Huus K. Participation of children with long-term health conditions compared to that of healthy peers: a cross-sectional comparative study. Scand J Occup Ther. (2022) 8:1–10. 10.1080/11038128.2022.2035815 35132920

[11] Kirk S Pryjmachuk S. Self-care of young people with long-term physical and mental health conditions. Nurs Child Young People. (2016) 28(7):20–8. 10.7748/ncyp.2016.e761 27615584

[12] Nilsson S Björkman B Almqvist AL Almqvist L Björk-Willén P Donohue D Children’s voices—differentiating a child perspective from a child’s perspective. Dev Neurorehabil. (2015) 18(3):162–8. 10.3109/17518423.2013.801529 23924164

[13] Shields N Synnot A. Perceived barriers and facilitators to participation in physical activity for children with disability: a qualitative study. BMC Pediatr. (2016) 16:1–10. 10.1186/s12887-016-0544-7 26786677 PMC4717582

[14] United Nations. Convention on the rights of the child. New York: UNICEF (1989).

[15] Dada S Andersson AK May A Andersson EE Granlund M Huus K. Agreement between participation ratings of children with intellectual disabilities and their primary caregivers. Res Dev Disabil. (2020) 104:103715. 10.1016/j.ridd.2020.103715 32574934

[16] Eiser C Morse R. Can parents rate their child’s health-related quality of life? Results of a systematic review. Qual Life Res. (2001) 10(4):347–57. 10.1023/a:1012253723272 11763247

[17] Büğüşan S Kahraman A Elbasan B Mutlu A. Do adolescents with cerebral palsy agree with their caregivers on their participation and quality of life? Disabil Health J. (2018) 11(2):287–92. 10.1016/j.dhjo.2017.10.009 29100958

[18] Cogan AM Carlson M. Deciphering participation: an interpretive synthesis of its meaning and application in rehabilitation. Disabil Rehabil. (2018) 40(22):2692–703. 10.1080/09638288.2017.1342282 28633542

[19] Mitra S Shakespeare T. Remodeling the ICF. Disabil Health J. (2019) 12(3):337–9. 10.1016/j.dhjo.2019.01.008 30910583

[20] Imms C Adair B Keen D Ullenhag A Rosenbaum P Granlund M. ‘Participation’: a systematic review of language, definitions, and constructs used in intervention research with children with disabilities. Dev Med Child Neurol. (2016) 58(1):29–38. 10.1111/dmcn.12932 26411643

[21] Adair B Ullenhag A Keen D Granlund M Imms C. The effect of interventions aimed at improving participation outcomes for children with disabilities: a systematic review. Dev Med Child Neurol. (2015) 57(12): 1093–104. 10.1111/dmcn.12809 26010935

[22] Imms C Granlund M Wilson PH Steenbergen B Rosenbaum PL Gordon AM. Participation, both a means and an end: a conceptual analysis of processes and outcomes in childhood disability. Dev Med Child Neurol. (2017) 59(1):16–25. 10.1111/dmcn.13237 27640996

[23] Adair B Ullenhag A Rosenbaum P Granlund M Keen D Imms C. Measures used to quantify participation in childhood disability and their alignment with the family of participation-related constructs: a systematic review. Dev Med Child Neurol. (2018) 60(11):1101–16. 10.1111/dmcn.13959 30022476

[24] Liao YT Hwang AW Liao HF Granlund M Kang LJ. Understanding the participation in home, school, and community activities reported by children with disabilities and their parents: a pilot study. Int J Environ Res Public Health. (2019) 16(12):2217. 10.3390/ijerph16122217 31238490 PMC6616950

[25] Gottlieb CA Maenner MJ Cappa C Durkin MS. Child disability screening, nutrition, and early learning in 18 countries with low and middle incomes: data from the third round of UNICEF’s multiple indicator cluster survey (2005–06). Lancet. (2009) 374(9704):1831–9. 10.1016/S0140-6736(09)61871-7 19944864

[26] Arvidsson P Dada S Granlund M Imms C Bornman J Elliott C Content validity and usefulness of picture my participation for measuring participation in children with and without intellectual disability in South Africa and Sweden. Scand J Occup Ther. (2020) 27(5):336–48. 10.1080/11038128.2019.1645878 31402722

[27] Samuels A Dada S Van Niekerk K Arvidsson P Huus K. Children in South Africa with and without intellectual disabilities’ rating of their frequency of participation in everyday activities. Int J Environ Res Public Health. (2020) 17(18):6702. 10.3390/ijerph17186702 32942532 PMC7558196

[28] Shi LJ Granlund M Zhao Y Hwang AW Kang LJ Huus K. Transcultural adaptation, content validity and reliability of the instrument ‘picture my participation’ for children and youth with and without intellectual disabilities in mainland China. Scand J Occup Ther. (2021) 28(2):147–57. 10.1080/11038128.2020.1817976 32941109

[29] Cameron L Murphy J. Enabling young people with a learning disability to make choices at a time of transition. Brit J Learn Disabil. (2002) 30(3):105–12. 10.1046/j.1468-3156.2002.00165.x

[30] Eiser C Morse R. A review of measures of quality of life for children with chronic illness. Arch Dis Child. (2001) 84(3):205–11. 10.1136/adc.84.3.205 11207164 PMC1718699

[31] Davis E Nicolas C Waters E Cook K Gibbs L Gosch A Parent-proxy and child self-reported health-related quality of life: using qualitative methods to explain the discordance. Qual Life Res. (2007) 16(5):863–71. 10.1007/s11136-007-9187-3 17351822

[32] Bendixen RM Senesac C Lott DJ Vandenborne K. Participation and quality of life in children with Duchenne muscular dystrophy using the international classification of functioning, disability, and health. Health Qual Life Outcomes. (2012) 10:43. 10.1186/1477-7525-10-43 22545870 PMC3358238

[33] Costa UM Brauchle G Kennedy-Behr A. Collaborative goal setting with and for children as part of therapeutic interventions. Disabil Rehabil. (2016) 16(39):1589–600. 10.1080/09638288.2016.1202334 27385635

[34] Huus K Granlund M Bornman J Lygnegård F. Human rights of children with intellectual disabilities: comparing self-ratings and proxy ratings. Child Care Health Dev. (2015) 41(6):1010–17. 10.1111/cch.12244 25809836

